# Mutual information-based teamwork evaluation in real-world environments: an exploratory investigation with professional surgeons

**DOI:** 10.3389/fnetp.2025.1608824

**Published:** 2025-09-04

**Authors:** Vincenzo Ronca, Lidia Castagneto Gissey, Maria Irene Bellini, Alessandra Iodice, Pietro Aricò, Gianluca Di Flumeri, Andrea Giorgi, Alessia Vozzi, Rossella Capotorto, Stefano Bonelli, Laura Moens, Fabio Babiloni, Giovanni Casella, Gianluca Borghini

**Affiliations:** ^1^ Department of Computer, Control, and Management Engineering “Antonio Ruberti”, Sapienza University of Rome, Rome, Italy; ^2^ BrainSigns srl, Rome, Italy; ^3^ Department of Surgery, Policlinico Umberto I, Sapienza University of Rome, Rome, Italy; ^4^ Department of Molecular Medicine, Sapienza University of Rome, Rome, Italy; ^5^ Department of Anatomical, Histological, Forensic & Orthopedic Sciences, Sapienza University of Rome, Rome, Italy; ^6^ DeepBlue srl, Rome, Italy; ^7^ Department of Computer Science, Hangzhou Dianzi University, Hangzhou, China; ^8^ Department of Physiology and Pharmacology “Vittorio Erspamer”, Sapienza University of Rome, Rome, Italy

**Keywords:** teamwork, mutual information, neurophysiological, human factors, electroencephalography, network physiology

## Abstract

**Purpose:**

Teamwork involves intricate interactions among individuals or groups with shared goals. It necessitates effective communication, defined roles, decision-making processes, and the allocation of cognitive and emotional resources. Objective teamwork assessment demands a comprehensive set of metrics. Although subjective and behavioral metrics, such as self-evaluation and task completion time, are generally applied, they are prone to bias and a lack of objectivity, highlighting the inherent limitations of capturing the unconscious processes of human behavior.

**Methods:**

To mitigate these limitations, the present study proposed a novel approach to teamwork evaluation based on neurophysiological signals (electroencephalograms, EEGs) compatible with real-world applications, i.e., surgical teams engaged in real-world surgeries. To the best of our knowledge, there is no scientific evidence of an objective teamwork measure performed among more than two members and relying on neurophysiological signals in real-world environments. Therefore, the present work aimed at i) developing and investigating the reliability of an objective EEG-based teamwork index using mutual information (MI) methods and ii) providing additional and objective insights for surgeons’ supervisors in healthcare training.

**Findings:**

The results demonstrated the capability of the EEG-based training index to provide additional and objective information, along with its added value and reliability compared to conventional measures (all R > 0.62, all *p* < 0.002). Furthermore, the EEG-based teamwork index allowed the determination (all *p* < 0.001) of surgeons’ experience levels (expert vs novice) in terms of cooperative behavior.

**Conclusion:**

The results pave the way for targeted interventions, adaptive training sessions, and optimizations in team dynamics and open up opportunities for applying neurophysiological measurements for teamwork evaluation in all operational fields, where proper and granular teamwork optimization could play a crucial role in terms of safety.

## 1 Introduction

In human factors (HF) research, cooperative behavior is studied using subjective, behavioral, and objective performance metrics. Subjective measurements, such as self-reports, and behavioral metrics, such as task completion time, are usually utilized. However, these approaches are susceptible to bias and a lack of objectivity. It is important to be aware of their inherent subjectivity and inability to capture the “unconscious” process underlying human behavior (Dienes et al., 2004; Borghini et al., 2016). To address these limitations, biometric and physiological monitoring emerges as a reliable and objective teamwork metric. Real-time monitoring of physiological responses, including brainwave patterns, cardiac activity, and skin conductance, provides insights into cognitive and emotional states; therefore, they can also offer an objective measure of team dynamics. Neurophysiological approaches to teamwork evaluation have been explored, with initial studies focusing on hyperscanning conditions for brain signal synchronization. Such a condition foresees the perfect synchronization among the different team members’ brain signals. Therefore, it is incompatible with real-world contexts and applications (Babiloni et al., 2006). In this regard, Astolfi et al. (2011) paved the way for the application of neurophysiological signal-based techniques in estimating brain synchronization within cooperative environments, while Toppi et al. (2016) extended this technique for characterizing cooperative behavior in ecological settings. Additionally, advances in electroencephalogram (EEG) signal processing, such as evaluating cortical connectivity using partial directed coherence (PDC), contributed to a nuanced understanding of cooperative environments. All the above-mentioned neurophysiology-based methods are characterized by a relevant limitation. First, they require the hyperscanning hypothesis (Koike et al., 2015), a neuroimaging technique that captures the synchronized neural activity between two or more individuals, which strictly requires perfect synchronization between the collected signals. More practically, hyperscanning requires collecting the EEG signals through the same EEG system. Network physiology, as an emerging interdisciplinary domain, investigates how physiological systems interact dynamically to sustain complex functions and states such as cognition, coordination, and performance. This paradigm goes beyond isolated measurements and focuses on cross-system communication and integration—for example, it examines how the brain, heart, and other organ systems synchronize and self-organize across time and context. Within this framework, cortical oscillations, especially functional brain connectivity, play a fundamental role in mediating coordinated cognitive and behavioral outcomes (Ivanov, 2021; Bashan et al., 2012). From this perspective, the cooperative behavior assessed in this study can be interpreted as a manifestation of multiscale network interactions across individuals’ brains operating within a shared task environment. The proposed mutual information (MI)-based approach aligns with this perspective by quantifying the shared information content between distributed neurophysiological states, thus representing a proxy for dynamic inter-brain coupling that supports the emergence of collective functions such as teamwork.

Since the above-mentioned hyperscanning-based approach cannot be applied in real-world environments, the present research relied on the MI technique (Steuer et al., 2002) to objectively assess the teamwork dynamics. This approach relies on the computation of shared information among different temporal series, which are, in the present study, associated with EEG spectral features. Typically, such features are characterized by a lower temporal dynamic than the EEG signal itself, and therefore, the MI technique helps mitigate the limitation imposed by the hyperscanning hypothesis. Such a method has already been explored in various studies for estimating cognitive load in cooperative environments (Rocha et al., 2005; Sciaraffa et al., 2017a; Sciaraffa et al., 2017b; Bezerianos et al., 2015). In the present study, the MI-based teamwork index was proposed and evaluated on surgeons’ teams composed of four members while performing a real-world surgery in the operating room.

In this regard, the assessment of teamwork in healthcare is increasingly recognized as an instrument to better understand human factors, enhance performance, and reduce errors (Foushee and Helmreich, 1988; Sexton et al., 2000; Saposnik et al., 2016; Wilson et al., 1996; Kurmann et al., 2012). In critical contexts, such as operating rooms, aircraft cockpits, air traffic control rooms, and nuclear power plants, efficient teamwork is essential for proper decision-making, safety, and the success of the mission. Although limited studies have assessed the impact of team training on patient outcomes, team training methods such as crew resource management (CRM) and team resource management (TMR) have demonstrated improved teamwork and reduced error rates (Ashcroft et al., 2021; JSTOR, 2023; Jakonen et al., 2023; Taylorfrancis, 2023). Salas’ model (Salas et al., 2018; Kozlowski et al., 2001) defines effective teamwork as comprising leadership, mutual performance monitoring, backup behavior, adaptability, and team orientation, supported by shared mental models, elements that are particularly crucial in healthcare. Although several approaches, including those based on MI (Zhao et al., 2024), have been proposed to evaluate cooperation between two team members, no existing methodology currently provides objective insights into shared mental models among more than two individuals using neurophysiological signals and multiple human factors simultaneously.

In the present study, the experimental design and analysis were designed according to specific hypotheses. In particular, i) it was assumed that experienced surgeons’ teams were more likely to cooperate, especially in real-world surgical environments; ii) in terms of conceptual neurophysiological characterization and as already proposed by Sciaraffa et al. (2021), it was assumed that the teamwork could be expressed as a combination of cognitive and affective surgeons’ states. Given this, the aim of the present study was two-fold: i) to develop and investigate the reliability of an objective teamwork index based on team members’ neurophysiological signals and ii) to provide supervisors with additional and objective insights to better evaluate operators’ expertise in healthcare training.

## 2 Materials and methods

### 2.1 Experimental sample and design

**FIGURE 1:**
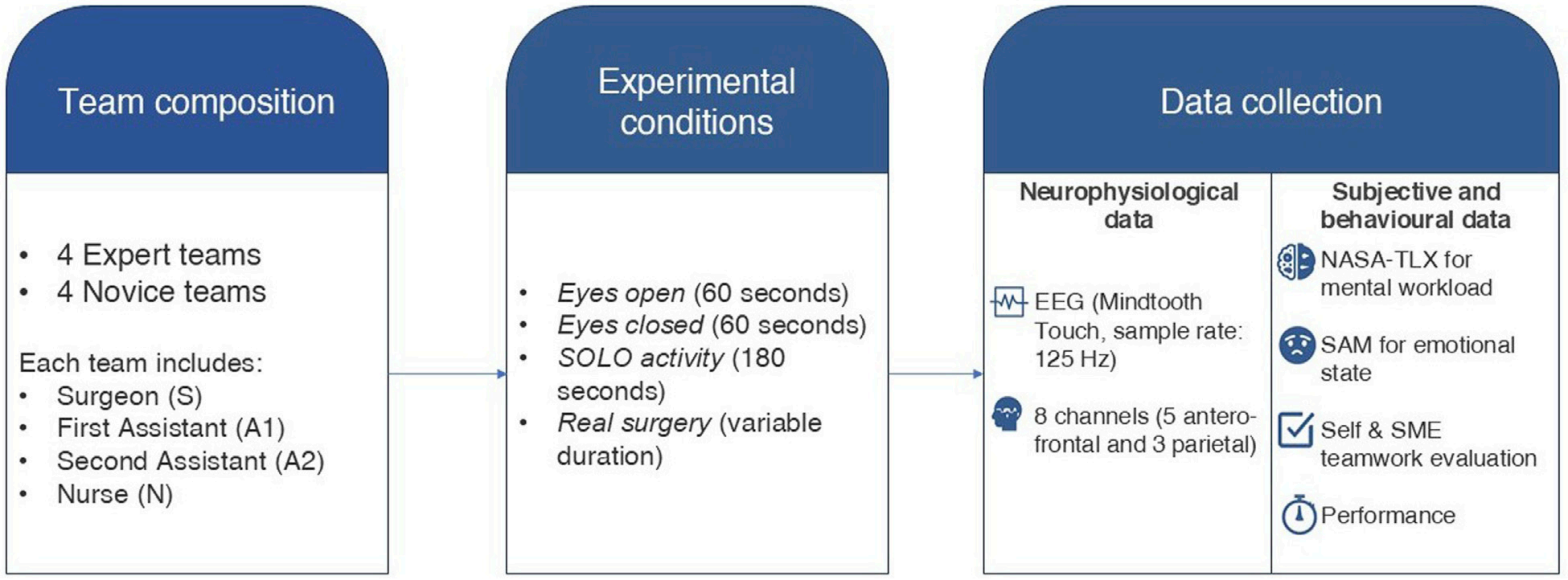
Summary of the experimental protocol design, including details about the experimental sample, conditions, and data collected.

Experiments were performed by recruiting surgeons from the Sapienza University Hospital of Rome, “Policlinico Umberto I” (Italy). Each team consisted of four members: an operating surgeon (S), a first assistant (A1), a second assistant (A2), and a scrub nurse (N). In particular, two categories of teams were defined based on the surgeon’s experience: *experts* and *novices*. The teams were asked to perform an inguinal hernia repair following the procedure described by Lichtenstein et al. (1989). This surgical procedure was also selected to allow novices to perform the operation. The expert group included surgeons who had performed at least 80 inguinal hernia repairs as the chief operating surgeon and were recognized by the National Specialist Register. Novice surgeons included residents actively training in a National General Surgery Residency Program. The criteria for recruitment in the novice group were set at a minimum of 3 years of residency and experience in hernia surgery. In particular, novice participants were required to have performed a minimum of 90 inguinal hernia repairs, with at least 40 inguinal hernia repairs as first assistant and 15 repairs as operating surgeon under the direct supervision of an expert surgeon. Surgeons from both groups were recruited from the Department of Surgical Sciences of the Sapienza University of Rome. A total of eight (8) surgical teams were involved in the experiment, equally divided between experts and novices. Experimental data were collected during real-world hernia surgeries, which were performed at the same time of day for all the teams. The experimental protocol consisted of three main phases: i) from skin incision to spermatic cord identification and isolation (*Phase 1*); ii) dissection of the hernia sac (*Phase 2*); and iii) mesh placement and fascia/skin closure (*Phase 3*). All the experimental design details are summarized in Figure 1. Given the exploratory nature of the present study, the discrimination between surgical phases was not examined in the statistical analysis. Baseline data were collected before surgery, consisting of 1 min with eyes open (required for eyeblink pattern recognition) and 1 min with eyes closed (condition for EEG frequency-band definition). The scrub nurse (N) performed a 3-min “SOLO condition,” preparing the instrument table, whereas at the end of the surgery, the other members (S, A1, and A2) were asked to individually suture a phantom for 3 min (SOLO condition). This step was required to compute the effective cooperation threshold for each member and the team and to normalize the data. The experiments were conducted in accordance with the principles of the Declaration of Helsinki (1975, revised in 2008) and were approved by the Ethical Committee of Sapienza University of Rome (protocol number: 0746/2023, approved on 20/09/2023). Informed consent was obtained from all the participants involved in the study, including the monitored surgical teams and patients. To respect the privacy of the surgeons, only aggregate results were reported, and no results based on single-identity analysis were presented, in accordance with the Regulation of the European Parliament 679/2016.

### 2.2 Subjective data

Subjective data were collected during the surgery by filling in various types of questionnaires. Such questionnaires were administered during and immediately after the end of the surgery to have a subjective assessment of teamwork and the emotional/cognitive states of the surgical team. More specifically, the subject matter expert (SME) filled in the questionnaires every 5 min, at the end of each phase of the surgery, and at the end of the experiment. Immediately after the end of the surgery, the team members completed a survey assessing the quality of collaboration with other members, their own performance, mental workload (MWL), stress, and emotions. All answers were collected on a 5-point Likert scale.

#### 2.2.1 Mental workload

The NASA-TLX questionnaire was used to measure the surgical teams’ perceived mental workload. This questionnaire divides mental workload into six subjective subscales: mental demand, physical demand, temporal demand, performance, effort, and frustration (Hart and Staveland, 1988). The questions were translated into the surgeons’ mother language (i.e., Italian), and the surgical team members rated their workload during the surgery on each of the six subscales. The final NASA-TLX score ranged from 0 to 100. A shortened version of this questionnaire was completed by the SME during the surgical observation, every 5 min. More specifically, the SME only rated the following statement: “*Evaluate the current workload of the operating team.*”

#### 2.2.2 Emotional state

The Self-Assessment Manikin (SAM) was used to measure the team members’ emotional states. The SAM is an inexpensive and easy method for quickly assessing affective responses in any context (Bynion and Feldner, 2020). The questionnaire consisted of three dichotomous scales, where each surgical team member had to indicate how much they felt (1) happy/unhappy, (2) excited/calm, and (3) controlled/in control. The same questionnaire was also completed by the SME. The questionnaire was completed at the end of the entire experimental protocol.

#### 2.2.3 Performance

Data on subjective performance were collected through direct questions at the end of the surgery. Surgical team members were asked to indicate both their individual and team performance (“*How successful were you in accomplishing what you were asked to do?*” and “*How adequate was the team’s performance?*”). The SME collected behavioral data related to the team performance; see Section 2.3. Both these scores ranged from 1 to 10.

As an additional indicator of performance, data on the patient outcome were collected through a questionnaire completed at the end of the surgery, in which the patients rated the physical and mental discomfort they experienced both during and immediately after the surgery. This score ranged from 1 to 5.

### 2.3 Behavioral data

The SME focused on several performance indicators while observing the surgical team. These indicators were determined at the beginning of the study in collaboration with SMEs to differentiate an adequate performance from an excellent performance. Every 5 min, the SME rated the speed of the team’s work, the harmoniousness of the surgeons’ movements, and the amount of bleeding. After the end of each of the three main phases, the SME recorded the number of materials used (i.e., fixators, retractors, forceps, needle holders, scissors, and gauzes). Finally, the SME evaluated how well the surgeons positioned the mesh. The completion time for the entire surgical session was recorded for each surgical team involved in this study.

### 2.4 Subjective and behavioral data combination

Subjective and behavioral data were combined to provide a more accurate evaluation of the team’s performance. In particular, two indices were defined: a combined subjective SME (cSME) and a combined behavioral teamwork index (CBTI). The cSME was defined as an index that combined all measures provided by the SME, thereby providing a composite representation of SME perceptions to be correlated with the neurophysiological teamwork index. More specifically, the cSME was obtained by linearly combining the SME mental workload and SME SAM evaluations (i.e., emotional-state self-evaluations provided by the surgeons’ team members), according to the initial hypothesis of this study— that the teamwork can be expressed as a combination of surgeons’ cognitive and affective states. Similarly, the CBTI was computed by linearly combining the normalized surgery time, the amount of surgical material used, and the patient outcome. Both the cSME and the CBTI were normalized across the set of surgical teams using the maximum and minimum values of their distributions. More specifically, the above-described indices were computed using Equations 1, 2:

$$cSME=\frac{1}{2}*{MWL}_{subjective}+\frac{1}{2}*SAM,$$ (1)

$$CBTI={\frac{1}{3}*Time}_{norm}+{\frac{1}{3}*Material}_{norm}+{\frac{1}{3}*Outcome}_{norm},$$ (2)

where ${MWL}_{subjective}$ represents the normalized subjective evaluation of the perceived mental workload provided by the surgeons; ${Time}_{norm}$ represents the normalized surgery time measured for each surgery intervention; ${Material}_{norm}$ represents the normalized amount of surgical material used for each intervention, and ${Outcome}_{norm}$ represents the normalized outcome evaluated by each patient involved in the surgery interventions (i.e., varying from 1 to $\frac{1}{5}$ ).

### 2.5 Neurophysiological data recording and processing

The surgeons’ EEG signals were collected using the digital monitoring system Mindtooth Touch (BrainSigns srl, Rome, Italy) at a sampling frequency of 125 Hz. During the experimental protocol, eight water-based recording electrodes were properly placed over the frontal and parietal scalp areas commonly identified for evaluating and assessing specific cognitive and mental states (Di Flumeri et al., 2022; Borghini et al., 2020; Ronca et al., 2022; Borghini et al., 2022; Di Flumeri et al., 2023). In particular, these EEG channels corresponded to the following channels: Afz, Af3, Af4, Af7, Af8, Pz, P3, and P4. These EEG channels were all referenced to the right mastoid and grounded to the left mastoid. Before starting the experimental data collection, the electrode impedances were verified to be below 50 kΩ (Sciaraffa et al., 2022) to guarantee adequate EEG signal quality.

Before starting the EEG signal analysis, a preprocessing phase was performed to identify and correct possible physiological and non-physiological content unrelated to the cerebral activity of interest (i.e., ocular, muscular, and movement-based artifacts). To this end, the EEG signal was band-pass-filtered using a fifth-order Butterworth filter in the interval of 2–30 Hz. The eye blink artifacts were detected and corrected online using the o-CLEAN method (Di Flumeri et al., 2016a; Ronca et al., 2024a). To address additional sources of artifacts, such as those caused by muscle activity and movement, *ad hoc* algorithms based on the EEGLAB toolbox (Delorme and Makeig, 2004) were applied. More specifically, two statistical criteria were applied to the 1-second-long preprocessed EEG signal. First, EEG epochs with signal amplitudes exceeding ±80 μV were marked as “artifacts.”’ Second, the EEG epochs marked as “artifacts” were removed from the EEG dataset to obtain an artifact-free EEG dataset.

Once the EEG preprocessing steps were completed, global field power (GFP) was calculated for the EEG frequency band of interest to evaluate the cognitive and mental states on which the present study focused. Therefore, the EEG GFP features were computed within the theta-, alpha-, and beta-frequency bands. It has to be underlined that the GFP was chosen as the parameter of interest describing brain EEG activity since it has the advantage of representing, within the time domain, the degree of synchronization on a specific cortical region of interest in a specific frequency band (Di Flumeri et al., 2016b; Skrandies, 1990). In terms of technical implementation, the GFP was mathematically computed according to the approach described by Vecchiato et al. (2014). Concerning the EEG GFP feature computation, the frequency bands were defined according to the individual alpha frequency (IAF) value (Klimesch, 1999) computed for each participant. To compute the IAF, a 60-second experimental condition was recorded while the participants kept their eyes closed, since the alpha peak is consistently prominent under these conditions. Subsequently, the EEG GFP was computed across all the EEG channels for each 1-second epoch using a Hanning window of the same length (1 s, which means 1 Hz of frequency resolution according to the time resolution required by the present approach). After EEG data preprocessing, the MI model was applied to define the EEG-based teamwork index. This procedure is described in the following sub-paragraph.

### 2.6 EEG-based teamwork index definition

The artifact-free EEG signals were processed to estimate the surgeons’ MWL and approach withdrawal (AW) using the same definitions used by Ronca et al. (2023), Di Flumeri et al. (2017), Giorgi et al. (2021), and Borghini et al. (2024). The assumption was that the surgeons’ teamwork could be modeled as the output of a multivariate system composed of the interaction between behavioral, affective, and cognitive mechanisms belonging to the surgical team members (Sciaraffa et al., 2021). The EEG-based teamwork index was obtained by computing the MI between the surgeons’ MWL and AW over 90-s time windows with a 1-s shift. In other words, the MI provides an objective indication of the maximum information shared between two random variables, including multivariate variables (Kraskov et al., 2004). As mentioned in the *Introduction*, such a concept does not rely on perfect signal synchronization, as hyperscanning-based applications do; instead, it allows capturing synchronization between human brains in terms of cognitive and affective states among more than two team members, which were observed within operational environments over time windows of several seconds (Borghini et al., 2020). Therefore, the MI assumptions were fully accomplished within the presented surgical scenario. Moreover, specific constraints were set on the MI model estimation to consider the real-world interactions among the team members during the three phases of the surgery (Steuer et al., 2002).

The selection of sub-teams (S–A1, S–N, A1–N, and A2–N) was guided by both operational and psychosocial considerations, as advised by expert surgeons from the “Policlinico Umberto I” Hospital. These pairings were not chosen arbitrarily but reflected key interaction dyads that are fundamental to the functional workflow and communication dynamics of the operating room.

• S–A1 (surgeon and first assistant): This pair represents the core surgical operators, with the first assistant playing a critical role in facilitating the primary procedure and closely supporting the surgeon.
• S–N (surgeon and nurse): This reflects the instrumental and coordination exchange as the nurse ensures that tools and materials are provided efficiently to the surgeon, impacting task fluidity and safety.
• A1–N (first assistant and nurse): This dyad captures instrument handling and backup behaviors, which are especially important in multi-instrument workflows.
• A2–N (second assistant and nurse): This pair was selected to represent peripheral but support-critical interactions that are often overlooked but essential for overall harmony and efficiency.

These sub-teams were selected to assess the degree to which mutual cognitive–affective states are synchronized in dyads with varying levels of task centrality, thus enabling a layered understanding of how expertise modulates teamwork within and beyond the core operative team.

#### 2.6.1 Neurometrics computation through the global field power

The use of GFP to characterize EEG activity is particularly relevant when considered within the emerging field of network physiology, which investigates how coordinated dynamics across multiple brain regions and physiological systems support functional states. GFP offers a compact representation of spatial synchronization in specific frequency bands and has been widely used to assess mental workload, attention, and affective states in applied contexts. Importantly, recent work in network physiology has demonstrated that synchronization and anti-synchronization patterns among cortical rhythms exhibit dynamic reorganizations that are highly state-dependent (Lin et al., 2020; Chen et al., 2022; Liu et al., 2015). These studies underscore that cross-frequency and spatial coupling among neural oscillations are not merely epiphenomena but are integral to adaptive regulation during transitions between rest, task engagement, and emotional processing. Thus, our use of GFP-based features to compute mental workload and approach–withdrawal aligns with this theoretical framework as it enables the capture of large-scale cortical interactions that reflect surgeons’ evolving cognitive and emotional states throughout the surgical task.

MWL and AW are considered to reflect the affective and cognitive mechanisms underlying human interactions, thereby supporting the definition of the teamwork index. In this regard, it should be noted that several previous scientific contributions proposed and validated the MWL evaluation as the frontal theta/parietal alpha ratio (Tao et al., 2019; Arico et al., 2014). A higher ratio (increased frontal theta and decreased parietal alpha) would suggest greater working memory engagement and mental effort. This aligns with the notion that demanding tasks require more cognitive resources, leading to increased frontal theta activity and reduced alpha activity as attentional processes are engaged (Ronca et al., 2024b; Klimesch, 2012; Hagemann aus Ebersberg et al., 2008). Similarly, concerning the AW computation, the use of frontal alpha asymmetry as an indicator of emotional valence and motivational tendencies, which are considered crucial components of effective teamwork, has been explored and validated in various previous scientific studies (Zhao et al., 2024; Borghini et al., 2023).

Therefore, the MWL and AW were computed by applying the GFP to the EEG frequency band of interest, according to previous studies (Skrandies, 1990). The GFP offers the advantage of representing the level of synchronization within a specific cortical region of interest in a particular frequency band in the time domain. Specifically, MWL and AW over time were calculated using Equations 3,4 [Disp-formula e4]:

$$MWL=\frac{{Theta}_{GFP}\left(Af3,Afz,Af4\right)}{A{lpha}_{GFP}\left(P3,Pz,P4\right)},$$ (3)

$$AW={Alpha}_{GFP}\left(Af4,Af8\right)-{Alpha}_{GFP}\left(Af3,Af7\right).$$ (4)

If any sample of the time series was missing due to the artifact rejection phase, the corresponding value was estimated by spline interpolation of the nearest epochs. Finally, each time series was z-score-normalized according to the entire distribution.

#### 2.6.2 EEG-based teamwork index computation through the mutual information technique

MI is an entropy-based index built on the concept that when two systems interact, mutual information can be derived from the interaction, and the entropy of the system decreases. MI thus quantifies the information shared between two or more time series, i.e., the EEG-based neurometrics (i.e., MWL and AW) derived from each team member. Therefore, MI allows discovering the maximum information shared between two random variables, including multivariate variables (Kraskov et al., 2004). In the scientific literature, previous works have demonstrated that entropy-based techniques, such as MI, are effective in quantifying the degree of shared information or coupling between time series. These methods are particularly useful for inter-brain or cross-participant analyses (Sauseng et al., 2005), such as team-based cognitive tasks, assessing how cognitive resources are shared during social interactions (Dum et al., 2010), and evaluating teamwork effectiveness in operational environments (Toppi et al., 2016; Babiloni et al., 2007). It is important to clarify that MI does not characterize the absolute brain network activity of individuals; rather, it captures statistical dependencies and concordances in cognitive–affective states (e.g., MWL and AW) across team members. Therefore, the observed entropy reduction reflects the similarity or alignment in the temporal evolution of these neurometrics among individuals, which we interpret as an indicator of teamwork-related synchrony, not as a standalone marker of network activity within a single brain. In the present study, each variable includes two different time series describing the affective and cognitive state of each team member. It has already been proven that there is a statistical relationship between teamwork effectiveness and the share of information between variables: higher mutual information values are associated with enhanced teamwork (Chicharro and Ledberg, 2012). The base formulations of this index are shown in Equations 5–8 as follows:

$$I\left(X,Y\right)\approx \sum_{i,j}p\left(i,j\right)\log\left(\frac{p\left(i,j\right)}{p_{x}\left(i\right)P_{y}\left(j\right)}\right),$$ (5)

$$p_{x}\left(i\right)=\int_{i}\mu_{x}\left(x\right)\;dx,$$ (6)

$$p_{y}\left(j\right)=\int_{j}\mu_{y}\left(y\right)\;dy,$$ (7)

$$p\left(i,j\right)=\iint \mu \left(x,y\right)dydx,$$ (8)

where $p\left(i,j\right)$ represents the joint probability, $p_{x}\left(i\right)$ and $p_{y}\left(j\right)$ represent the marginal probabilities, $\mu_{x}$ and $\mu_{y}$ represent the probability densities, and i and j represent the bin of the discrete 2D space bounded by the two time series (i.e., neurometrics included in the MI model).

Formula 8 is already an approximation of the continuous MI evaluated on a continuous space. This formulation was technically implemented to discretize the signal probability density evaluation, and it is conceptually developed as total correlation (Watanabe, 1960). In this regard, Kraskov et al. (2004) proposed an estimator of MI based on the entropy concept (Equation 9) (Kraskov et al., 2004):

$$Teamwork\;Index=I\left(X_{1},X_{2},\ldots ,X_{m}\right)=H\left(X_{1}\right)+H\left(X_{2}\right)+...\;H\left(X_{m}\right)-H\left(X_{1},X_{2},\ldots ,X_{m}\right).$$ (9)

This estimator corresponds to a KNN-based method (Li et al., 2011), which evaluates $H\left(X_{i}\right)$ from the average distance of the *k*th nearest neighbor, averaged over all other points of the signal. More specifically, the addends included in Formula 9 represent the entropy-based MI associated only with each time series (i.e., $X_{1},X_{2},\ldots ,X_{m}$ in the proposed example). The time series considered for the presented teamwork index computation corresponded to the above-described MWL and AW features. Such an approach was already successfully proposed for a different application in a similar out-of-the-laboratory context (Ronca et al., 2025).

### 2.7 Statistical analysis

Data were normalized before starting the statistical analyses. More specifically, the subjective and behavioral data, including the cSME and CBTI scores, were normalized by scaling them based on their individual maximum and minimum values across the entire experimental session and for each surgical team. Meanwhile, the EEG features, i.e., the EEG-based teamwork index, were subjected to normalization through the application of the z-score approach, referencing the data series to the whole experimental session. As a preliminary step, the Shapiro–Wilk test (González-Estrada et al., 2022) was selected to determine the normality of the distribution related to each of the considered statistical features. In the case of normal distributions, the Student’s t-test was selected for comparing the two experimental groups (i.e., experts vs novices). If the normality of distributions was not confirmed using the Shapiro–Wilk test, the Mann–Whitney test was performed. Additionally, the repeated-measures correlation analysis (Bakdash and Marusich, 2017) was performed to validate the EEG-based teamwork index with respect to the subjective and behavioral measurements collected from the surgical team, both at the single-participant level and in the entire group.

## 3 Results

The results were organized into three different subsections, namely, subjective and behavioral measures, EEG-based teamwork assessment, and EEG-based teamwork index validation, to facilitate readability. All the results are shortly described in these subsections, pointing out the relevant findings, while they are discussed in a more comprehensive view in the *Discussion* section.

### 3.1 Subjective and behavioral measures

The statistical analysis performed on the cSME did not reveal any statistical difference (*p* = 0.27; Cohen’s d = 0.102) between the expert and novice surgical teams in terms of team cooperative dynamics perception provided by the SME.

**FIGURE 2:**
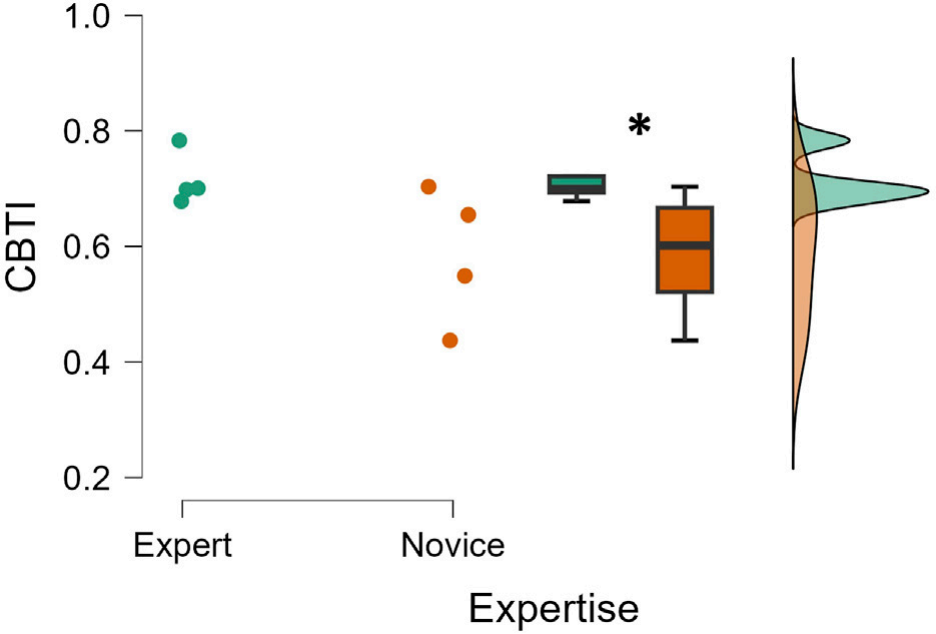
The Mann–Whitney test performed on the CBTI parameter revealed that the expert surgical teams exhibited higher performance than the novice teams (*p* = 0.01). Each dot represents the average index across the entire surgery for one of the eight teams (n = 8; 4 experts and 4 novices).

On the contrary, the Mann–Whitney test performed on the CBTI (Figure 2) revealed a statistical difference (*p* = 0.01; Cohen’s d = 0.870) between the two considered surgical team groups. In particular, it was observed that the expert surgical teams exhibited a statistically higher performance than the novice teams.

### 3.2 EEG-based teamwork index assessment

**FIGURE 3:**
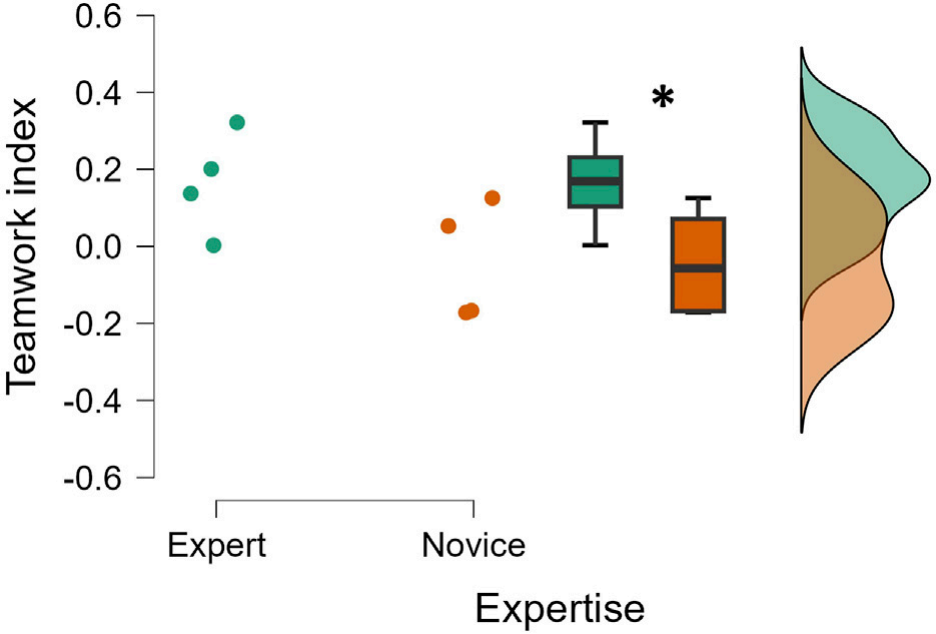
The EEG-based teamwork index was significantly higher for expert teams than for novice teams (*p* = 0.02, Cohen’s d = 0.889). Each dot represents the average index across the entire surgery for one of the eight teams (n = 8; 4 experts and 4 novices).

The statistical analysis performed on the EEG-based teamwork index demonstrated that the expert surgical group showed a statistically higher (*p* = 0.02; Cohen’s d = 0.889) level of teamwork during the entire surgery than the novice group (Figure 3).

### 3.3 Expertise impact on teamwork hub

**FIGURE 4:**
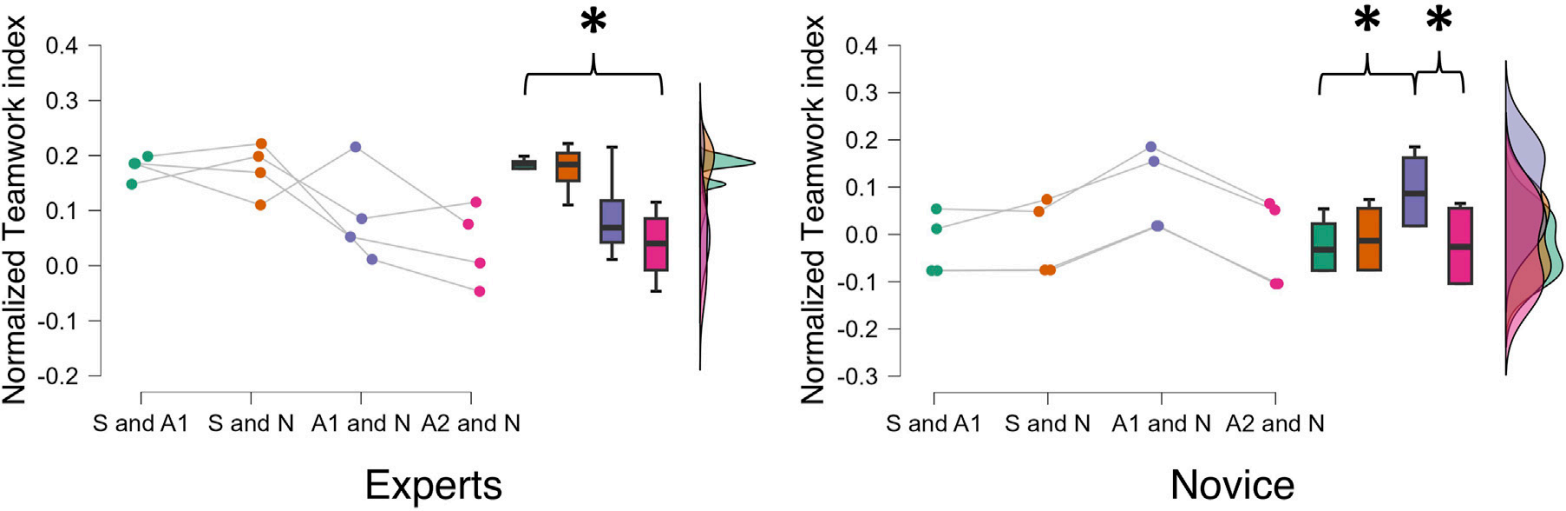
The statistical analysis performed on the surgical sub-teams (i.e., S–A1, S–N, A1–N, and A2–N) demonstrated how the proposed EEG-based teamwork index allowed to statistically discriminate between expert and novice surgeons’ teams, highlighting that the major cooperative dynamics were identified through the EEG-based teamwork index among the sub-teams, in which the experienced member was operating (i.e., the S–A1 for expert and A1–N for novice). Each dot represents the average index across the entire surgery for the considered surgical couple (n = 32; 16 expert couples and 16 novice couples).

Interestingly, the statistical analysis performed on the EEG-based teamwork index computed among the sub-teams (i.e., S–A1, S–N, A1–N, and A2–N) mentioned previously revealed a significant difference between the expert and novice surgical teams (F = 6.716, *p* = 0.03, and η^2^ = 0.401). In particular, the sub-team analysis demonstrated that the expert surgeons served as the teamwork hub even in the novice teams despite their role as A1 (F = 8.031, *p* = 0.001, and η^2^ = 0.509). In this regard, Figure 4 shows how the expert sub-team S–A1 exhibited a higher teamwork degree than the A2–N sub-team (*p* = 0.04; η^2^ = 0.159), while the novice sub-team A1–N showed higher teamwork than the S–A1 and A2 – N sub-teams (*p* = 0.02; η^2^ = 0.227).

### 3.4 EEG-based teamwork index validation

**FIGURE 5:**
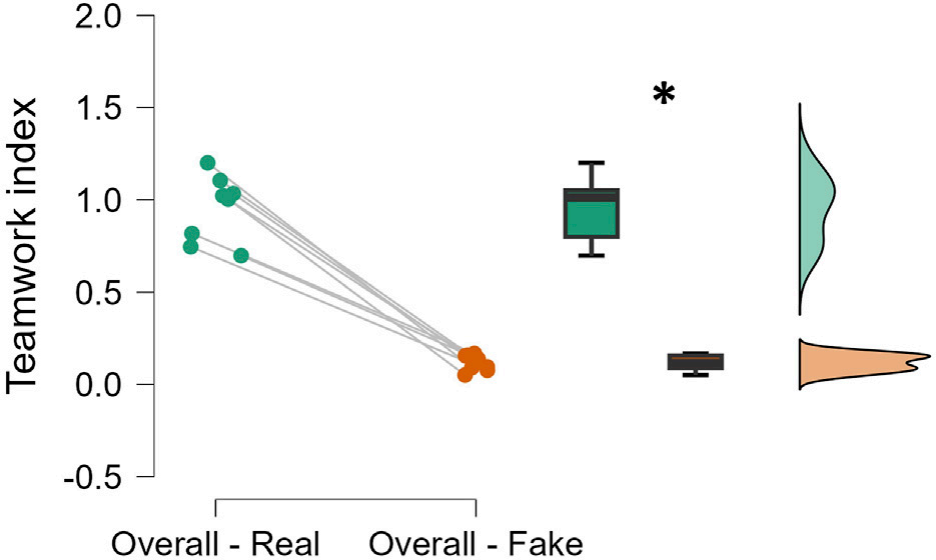
The statistical analysis performed on the EEG-based teamwork index demonstrated its reliability for detecting real-world human brain synchronization in terms of operational teamwork (i.e., real-world surgical teams) compared to the teamwork associated with simulated surgical teams (i.e., fake) (*p* = 0.0001; η^2^ = 0.689).

To validate the specificity of the EEG-based teamwork index, a control analysis was conducted comparing real-world surgical teams (i.e., *real-world* teams) with randomly composed “fake” teams (i.e., *fake* teams), i.e., EEG signals from individuals participating in different surgical procedures and who did not collaborate. This analysis was designed to ensure that the MI-based model is sensitive to actual inter-brain interaction resulting from shared task engagement, rather than to spurious statistical dependencies across unrelated signals. It must be emphasized that the signal portions being compared (i.e., those from the real-world teams and those from the fake teams) were selected at the same specific time point during the surgical procedure. As shown in Figure 5, the teamwork index was significantly higher for real-world teams than for fake teams (*p* = 0.0001; η^2^ = 0.689), where MI values approached 0. This result confirms that the model captures meaningful coupling related to cooperative cognitive and emotional processes and not random background synchrony. Such surrogate comparisons are widely adopted in hyperscanning and inter-brain studies as a standard validation step to verify that observed synchrony arises from true social interaction rather than common task structure or shared stimulus processing (Czeszumski et al., 2020; Dum et al., 2010).

**FIGURE 6:**
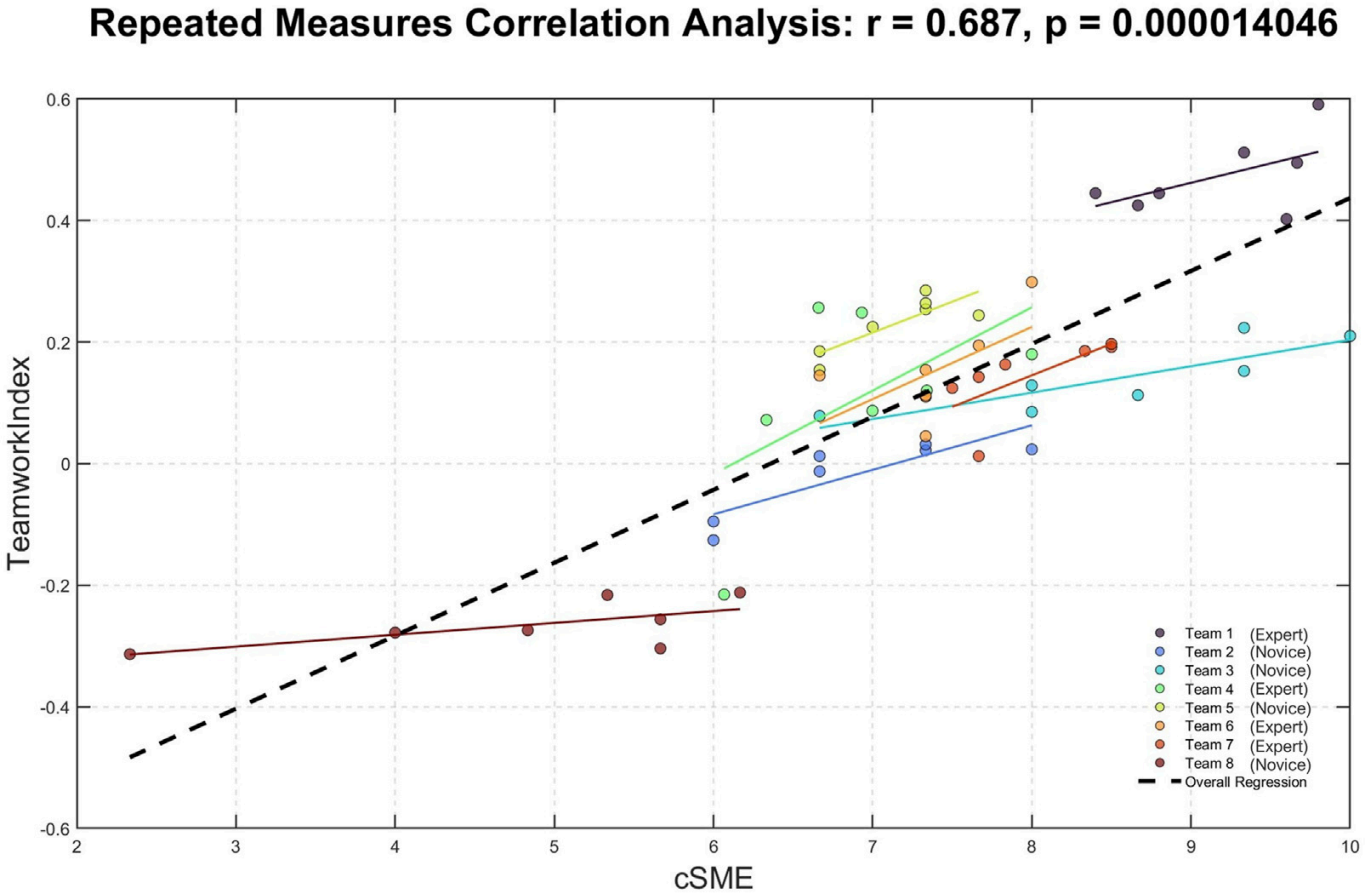
The repeated-measures correlation analysis demonstrated that the EEG-based teamwork index was significantly and positively correlated (R = 0.68; *p* < 0.0001) with the teamwork evaluations provided by the SME.

**FIGURE 7:**
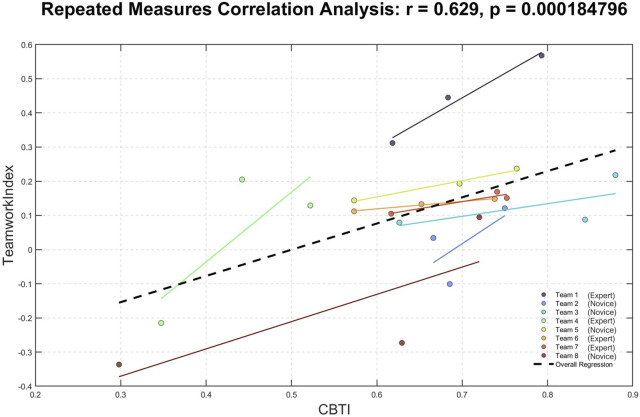
The repeated-measures correlation analysis demonstrated that the EEG-based teamwork index was significantly and positively correlated (R = 0.63; *p* = 0.0002) with the surgeons’ performance.

To further support the validity of the EEG-based teamwork index, we investigated its relationship with independently collected subjective and behavioral assessments of teamwork. Specifically, we performed repeated-measures correlation analyses between the EEG-based index and two composite parameters: the combined subjective measure provided by the SME (cSME) and the CBTI. The results demonstrated significant positive correlations in both cases (cSME: R = 0.69, *p* < 0.0001; CBTI: R = 0.63, p = 0.0002), confirming that higher values of the EEG-based index were associated with improved teamwork evaluations by experts and superior behavioral performance metrics (Figures 6, 7). These findings reinforce the reliability of the neurophysiological index as an objective measure of team dynamics during surgery.

## 4 Discussion

The present study had two objectives. First, it introduced and validated an EEG-based teamwork index designed to quantify cooperative dynamics among surgical team members in real-world operating room conditions. Second, it assessed the reliability of this neurophysiological measure as an objective tool to support supervisors in evaluating individual surgeons’ teamwork capabilities during training. Notably, the focus was not on assessing team performance as a static group entity but rather on capturing each individual’s ability to engage in effective team interactions. This is extremely coherent with the dynamics characterizing real-world healthcare environments, especially in surgery, where surgical team compositions frequently change and consistent team membership is rarely feasible.

Regarding the first objective of the study, repeated-measures correlation analysis highlighted how the EEG-based teamwork index was consistently and significantly correlated with the supervisor teamwork evaluations, the surgeons’ performance, and the surgeons’ teamwork perceptions in the experimental task (i.e., the entire surgery) (Figures 6, 7). Such results are fully coherent with the experimental hypothesis. In addition to the positive and significant correlation with the subjective ratings related to the teamwork perception (cSME-combined parameter), it has to be noted that the surgeons’ performance along the surgery (CBTI-combined parameter) is positively and significantly correlated with the teamwork objective evaluation, as supported by several previous works, in which it was demonstrated how consistent teamwork among surgical team members increases their performance (Etherington et al., 2021). These findings support the initial hypothesis that cooperative behavior during surgery is reflected in synchronized cognitive and affective states among team members, which can be objectively quantified using EEG-derived measures.

Regarding the second objective of the study, the independent pairwise comparisons between experts and novices highlighted how an objective teamwork evaluation can provide consistent and unique insights to supervisors in healthcare training programs, which are known to be based primarily on experience in real-world contexts, as reproduced in the experimental protocol presented in this study (Eppich et al., 2011; Cifalino and Baraldi, 2009; Omura et al., 2017). By applying the above-described neurophysiological approach, it was possible to discriminate between two different categories in terms of surgical experience (Figure 3). More importantly, statistical analysis performed on the surgical sub-teams (S–A1, S–N, A1–N, and A2–N) as defined according to the surgeons’ hypothesis in the experimental design revealed that the EEG-based teamwork index was statistically higher when evaluated among the sub-teams containing an experienced member, i.e., the S–A1 for the expert group and the A1–N for the novice group (Figure 4). This result confirmed the sensitivity of the proposed EEG-based teamwork index in detecting different levels of cooperative behavior among operators with different expertise, consistent with previous works showing that expertise is consistently associated with a higher grade of cooperative dynamics (Chu and Hwang, 2008). Finally, repeated-measures correlations, along with the results of the independent pairwise comparisons, revealed the crucial sensitivity of the proposed EEG-based teamwork index in discriminating between two experience-based surgical team categories (i.e., expert and novice). Although subjective evaluations provided by the SME (cSME) did not show statistically significant differences between expert and novice teams, the EEG-based teamwork index revealed a significant distinction between these two groups (Figure 3). This suggests that the neurophysiological approach was more sensitive in capturing subtle variations in cooperative behavior associated with surgical expertise under the experimental conditions of this study. It is important to clarify that this distinction is based on empirical evidence from the data rather than a theoretical assumption about the superiority of neurophysiological metrics. The finding highlights the added value of objective, brain-based measures in complementing traditional observational assessments within training contexts.

Moreover, the outcomes of the present study can be interpreted through the lens of network physiology, which considers physiological and cognitive processes to be emergent from interdependent and interacting subsystems. In this study, mutual information computed across multiple team members’ EEG-derived neurometrics (mental workload and approach–withdrawal) reflects the degree of informational coupling among the cortical subsystems engaged in joint task execution. In particular, these two neurometrics were specifically chosen to represent complementary aspects of teamwork: MWL reflects the cognitive subsystem, where high synchrony may indicate a balanced distribution of mental effort across team members; conversely, AW reflects the affective subsystem, where coherence in approach-oriented signals among participants may suggest that the team is not only cognitively aligned but also emotionally attuned and positively engaged in the collaborative effort. By avoiding strict hyperscanning constraints, our method captures functional brain-to-brain interactions that evolve naturally in real-world environments. This aligns with the network physiology perspective, emphasizing that cognitive functions such as attention, decision-making, and teamwork emerge from distributed, context-sensitive system-level interactions rather than isolated node activity. Thus, the proposed EEG-based teamwork index contributes to the understanding of how inter-brain communication supports adaptive regulation of shared physiological and cognitive states in complex environments such as an operating room.

From a broader perspective, the present findings contribute to the literature on network-level brain dynamics that support cognitive coordination during complex real-world tasks. The use of GFP-derived neurometrics, specifically in the theta and alpha bands, allowed us to estimate the degree of cortical synchrony associated with attentional and emotional processes. This approach is consistent with recent advances in network physiology that have emphasized the role of dynamic and multiscale interactions among brain rhythms across physiological states such as sleep, exercise, and cognitive engagement (Lin et al., 2020; Chen et al., 2022; Liu et al., 2015). These interactions represent a physiological substrate for the emergence of higher-order functions such as cooperative decision-making and performance adaptation—precisely the types of functions being assessed in surgical teams. In this light, future work could enhance the robustness of our teamwork evaluation framework by exploring complementary measures of dynamic connectivity. For instance, cross-correlation and time-delay stability metrics have been shown to capture the temporal dependencies and the directionality of interactions across spatially distributed signals. These measures could be integrated with or used in place of mutual information to assess how reliably and flexibly team members’ neurophysiological states co-evolve during high-stakes tasks. Such extensions would further align the current method with the goals of network physiology by emphasizing real-time, dynamic coupling patterns that reflect the system’s capacity for self-organization under cognitive and emotional demands.

### 4.1 Limitations

Despite the positive results, several limitations should be noted. For instance, the sample size was not large. One important methodological consideration concerns the use of the KNN-based estimator for computing mutual information. Although this estimator is widely used due to its non-parametric nature and robustness in many contexts, it is sensitive to data properties such as non-stationarity, noise, and long-range correlations—particularly in the context of physiological signals such as EEG (Xiong et al., 2017). These properties can lead to the over- or underestimation of shared information content, thereby impacting the reliability of the EEG-based teamwork index. In our study, several preprocessing steps were taken to mitigate such issues, including artifact removal, z-score normalization, and temporal segmentation. Nonetheless, we acknowledge that some degree of non-stationarity may persist, especially in real-world surgical environments where physiological and contextual variabilities are inherently high. Future work may benefit from exploring complementary estimators—such as adaptive binning, symbolic transfer entropy, or time-delay mutual information—that are potentially more resilient to these effects. Additionally, assessing local stationarity and spectral stability within time windows could offer further validation of MI-based measures in dynamic operational tasks. Additionally, the study focused on a single surgical procedure (inguinal hernia repair), which may not fully represent the complexity and variability of teamwork dynamics across different surgical specialties. Expanding the research to include various surgical procedures would provide a more comprehensive understanding of teamwork in the operating room. It should be noted that the present study represents an initial investigation and validation of the proposed technique for objective teamwork evaluation, and more importantly, it was performed in real-world environments, while the surgical teams were performing a real-world surgery. The surgeons’ recruitment and the experimental settings proved to be highly challenging in terms of logistical and legal requirements. Future research should focus on investigating a larger sample size, different surgical contexts, and incorporating a more fine-grained analysis to characterize the temporal modulation of the teamwork evaluation. In terms of the practicality of the proposed approach, the integration of EEG-based assessments into high-stakes, real-time surgeries may not yet be possible for routine use; the growing availability of wearable and minimally invasive EEG systems opens the possibility for such tools to be used in simulation-based training or post-operative evaluation contexts. This study serves as a proof-of-concept demonstrating that EEG-derived neurometrics can reliably reflect teamwork quality, offering a foundation for future developments in neuroergonomic evaluation methods. Finally, future approaches could consider a further validation of the proposed technique for the teamwork evaluation by analyzing its co-variation with other physiological features, such as those derived from the electrodermal and cardiac activities.

## 5 Conclusion

This study presents a promising approach for objectively assessing teamwork using EEG data in a real-world surgical setting. The developed EEG-based teamwork index demonstrated significant correlations with subjective and behavioral measures, highlighting its reliability and potential for providing valuable insights into team dynamics. The ability to differentiate between expert and novice surgical teams further underscores its utility in healthcare training and performance evaluation. Furthermore, the present study was conducted in a real -world surgical environment, and this aspect consistently contributes to its novelty. To the best of our knowledge, no similar scientific works exist in the literature for objective teamwork quantification, specifically evaluating teamwork among more than two cooperating team members. The results open up opportunities for targeted interventions, training programs, and improvements in team dynamics and pave the way for applying the neurophysiological approach for the teamwork evaluation in similar and wider operational fields, where proper and granular teamwork optimization could play a crucial role in terms of safety.

## Data Availability

The datasets presented in this article are not readily available because the generated data could not be fully anonymized. Requests to access the datasets should be directed to vincenzo.ronca@uniroma1.it.
